# (9*S*,13*R*,14*S*)-7,8-Didehydro-4-(4-fluoro­benz­yloxy)-3,7-dimeth­oxy-17-methyl­morphinan-6-one sesquihydrate

**DOI:** 10.1107/S1600536811026183

**Published:** 2011-07-09

**Authors:** Xing-Liang Zheng, Ning-Fei Jiang, Dan Luo, Hong-Sheng Gao, Ai-Shun Ding

**Affiliations:** a School of Chemistry and Biological Engineering, Changsha University of Science & Technology, Changsha 410114, People’s Republic of China

## Abstract

In the title sinomenine derivative, C_26_H_28_FNO_4_·1.5H_2_O, the dihedral angle between the two aromatic rings is 55.32 (6)°. The N-containing ring has an approximate chair conformation, while other two rings have approximate envelope and half-chair conformations. One water mol­ecule is located on a twofold symmetry axis. In the crystal, the water mol­ecules form O—H⋯O and O—H⋯N hydrogen bonds, bridging symmetry-related main mol­ecules.

## Related literature

For background to the biological activity of sinomenine derivatives and other related compounds, see: Liu *et al.* (1994 ▶, 1996 ▶, 1997 ▶); Mark *et al.* (2003 ▶); Ye *et al.* (2004 ▶). For the synthesis of the title compound, see: Mitsunobu (1981 ▶). For related structures, see: Li *et al.* (2009 ▶); Batterham *et al.* (1965 ▶); Zheng & Jiang (2010 ▶); Zheng *et al.* (2011 ▶).
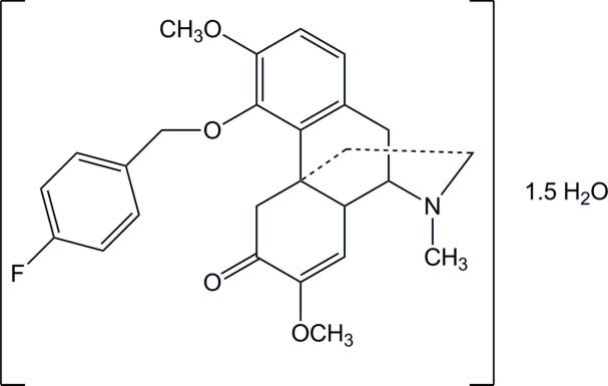


## Experimental

### 

#### Crystal data

C_26_H_28_FNO_4_·1.5H_2_O
*M* *_r_* = 464.52
Monoclinic, 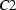

*a* = 18.0155 (3) Å
*b* = 7.6776 (1) Å
*c* = 18.1506 (4) Å
β = 109.324 (1)°
*V* = 2369.08 (7) Å^3^
*Z* = 4
Cu *K*α radiation
μ = 0.79 mm^−1^
*T* = 133 K
0.25 × 0.15 × 0.10 mm

#### Data collection

Bruker APEXII CCD diffractometer
Absorption correction: multi-scan (*SADABS*; Bruker, 2000 ▶) *T*_min_ = 0.826, *T*_max_ = 0.925
7232 measured reflections
3415 independent reflections
3402 reflections with *I* > 2σ(*I*)
*R*_int_ = 0.024

#### Refinement

*R*[*F* ^2^ > 2σ(*F* ^2^)] = 0.028
*wR*(*F* ^2^) = 0.081
*S* = 1.04
3415 reflections
312 parameters
1 restraint
H atoms treated by a mixture of independent and constrained refinement
Δρ_max_ = 0.20 e Å^−3^
Δρ_min_ = −0.18 e Å^−3^
Absolute structure: Flack (1983 ▶), 1359 Friedel pairs
Flack parameter: 0.05 (12)

### 

Data collection: *APEX2* (Bruker, 2000 ▶); cell refinement: *SAINT* (Bruker, 2000 ▶); data reduction: *SAINT*; program(s) used to solve structure: *SHELXS97* (Sheldrick, 2008 ▶); program(s) used to refine structure: *SHELXL97* (Sheldrick, 2008 ▶); molecular graphics: *SHELXTL* (Sheldrick, 2008 ▶); software used to prepare material for publication: *SHELXTL* (Sheldrick, 2008 ▶).

## Supplementary Material

Crystal structure: contains datablock(s) global, I. DOI: 10.1107/S1600536811026183/bh2361sup1.cif

Structure factors: contains datablock(s) I. DOI: 10.1107/S1600536811026183/bh2361Isup2.hkl

Additional supplementary materials:  crystallographic information; 3D view; checkCIF report

## Figures and Tables

**Table 1:** Hydrogen-bond geometry (Å, °)

*D*—H⋯*A*	*D*—H	H⋯*A*	*D*⋯*A*	*D*—H⋯*A*
O2*S*—H22*S*⋯O4	0.91	2.33	2.9805 (16)	128
O2*S*—H22*S*⋯O3	0.91	2.54	3.417 (2)	164
O1*S*—H11*S*⋯O2*S*	0.90 (3)	1.94 (3)	2.8342 (19)	172 (2)
O2*S*—H21*S*⋯N1^i^	0.97	1.81	2.7736 (19)	170

Symmetry code: (i) 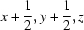.

**Fig. 1.**
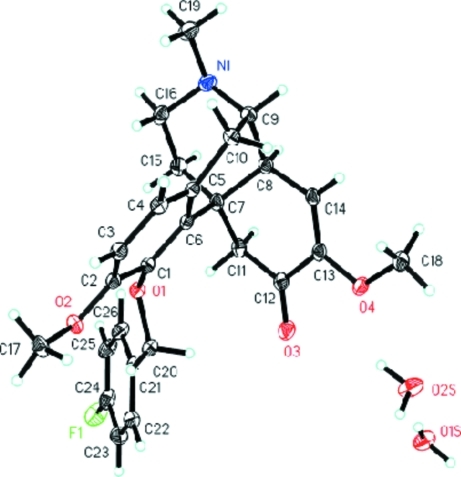
The molecular structure of the title compound showing 50% probability displacement ellipsoids.

**Fig. 2.**
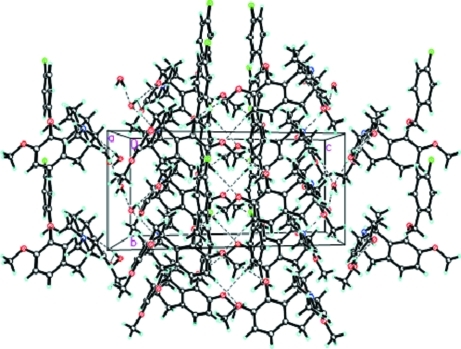
A part of the crystal structure of the title compound.

## supplementary crystallographic information

### Comment

We synthesized a new sinomenine derivative sesquihydrate. Herein, its crystal structure is reported. Biological effects of sinomenine derivatives and related compounds have been described (Liu *et al.*, 1994, 1996, 1997; Mark *et al.*, 2003; Ye *et al.*, 2004).

The molecular structure of the title compound is shown in Fig. 1. The crystal structure is stabilized by O—H···O and O—H···N hydrogen bonds linking the sinomenine derivative and the water molecules, and weak C—H···O hydrogen bonds between molecules (Fig. 2). Significant aromatic stacking interactions were not found. There exist two planes in the molecule of the title compound: atoms C1···C6 form the benzene plane (I), and atoms C21···C26 form the benzene plane substituted by fluorine (II). The angle between the two planes (I) and (II) is 55.32 (6)°. Rings *C* [C7/C8/C11/C12/C13/C14] and *B* [C5···C10] in the molecule approximate envelope and half-chair conformations, respectively. In contrast, ring *D* [C9/N1/C16/C15/C7/C8] exhibits an almost regular chair conformation. Similar features have been described in related compounds (Zheng & Jiang, 2010; Zheng *et al.*, 2011; Li *et al.*, 2009; Batterham *et al.*, 1965).

### Experimental

The title compound was obtained according to the method of Mitsunobu (1981). Colorless blocks were grown from an acetic ether solution.

### Refinement

The water H atoms (H21S, H22S and H11S) were located in a difference map. H11S was refined with free coordinates and isotropic displacement parameter. H21S and H22S were fixed in their as found positions and allowed to ride on O2S, and their displacement parameters were refined. Other H atoms were positioned geometrically, with C—H = 0.95 (aromatic CH), 0.98 (methyl CH_3_), 0.99 (methylene CH_2_) or 1.00 Å (methine CH), and were constrained to ride on their parent atoms, with *U*_iso_(H) = 1.2*U*_eq_(carrier C) or *U*_iso_(H) = 1.5*U*_eq_(carrier C17 C18 C19). 1359 Friedel pairs were used for the Flack parameter refinement.

### Crystal data

**Table:**

C_26_H_28_FNO_4_·1.5H_2_O	*F*(000) = 988
*M**_r_* = 464.52	*D*_x_ = 1.302 Mg m^−^^3^
Monoclinic, *C*2	Cu *K*α radiation, λ = 1.54178 Å
Hall symbol: C 2y	Cell parameters from 6582 reflections
*a* = 18.0155 (3) Å	θ = 2.6–64.7°
*b* = 7.6776 (1) Å	µ = 0.79 mm^−^^1^
*c* = 18.1506 (4) Å	*T* = 133 K
β = 109.324 (1)°	Block, colourless
*V* = 2369.08 (7) Å^3^	0.25 × 0.15 × 0.10 mm
*Z* = 4	

### Data collection

**Table:**

Bruker APEXII CCD diffractometer	3415 independent reflections
Radiation source: fine-focus sealed tube	3402 reflections with *I* > 2σ(*I*)
graphite	*R*_int_ = 0.024
φ and ω scans	θ_max_ = 65.0°, θ_min_ = 2.6°
Absorption correction: multi-scan (*SADABS*; Bruker, 2000)	*h* = −20→21
*T*_min_ = 0.826, *T*_max_ = 0.925	*k* = −9→9
7232 measured reflections	*l* = −18→20

### Refinement

**Table:**

Refinement on *F*^2^	Secondary atom site location: difference Fourier map
Least-squares matrix: full	Hydrogen site location: inferred from neighbouring sites
*R*[*F*^2^ > 2σ(*F*^2^)] = 0.028	H atoms treated by a mixture of independent and constrained refinement
*wR*(*F*^2^) = 0.081	*w* = 1/[σ^2^(*F*_o_^2^) + (0.0555*P*)^2^ + 0.6114*P*] where *P* = (*F*_o_^2^ + 2*F*_c_^2^)/3
*S* = 1.04	(Δ/σ)_max_ < 0.001
3415 reflections	Δρ_max_ = 0.20 e Å^−^^3^
312 parameters	Δρ_min_ = −0.18 e Å^−^^3^
1 restraint	Absolute structure: Flack (1983), 1359 Friedel pairs
0 constraints	Flack parameter: 0.05 (12)
Primary atom site location: structure-invariant direct methods	

### Fractional atomic coordinates and isotropic or equivalent isotropic displacement parameters (Å^2^)

**Table:**

	*x*	*y*	*z*	*U*_iso_*/*U*_eq_	
F1	0.49485 (7)	−0.26627 (16)	0.40332 (8)	0.0571 (3)	
N1	−0.03356 (8)	0.4513 (2)	0.15607 (8)	0.0314 (3)	
O1	0.26142 (6)	0.36402 (13)	0.34083 (6)	0.0237 (2)	
O2	0.29280 (6)	0.57951 (14)	0.46321 (6)	0.0268 (2)	
O3	0.32140 (7)	0.48228 (18)	0.15878 (7)	0.0392 (3)	
O4	0.24651 (6)	0.74481 (15)	0.07306 (7)	0.0307 (3)	
C1	0.21885 (8)	0.51681 (19)	0.33202 (9)	0.0217 (3)	
C2	0.23288 (8)	0.6279 (2)	0.39717 (9)	0.0233 (3)	
C3	0.18682 (9)	0.7742 (2)	0.39246 (9)	0.0265 (3)	
H3	0.1968	0.8511	0.4357	0.032*	
C4	0.12611 (8)	0.80691 (19)	0.32395 (9)	0.0253 (3)	
H4	0.0943	0.9072	0.3209	0.030*	
C5	0.10990 (8)	0.6993 (2)	0.25971 (9)	0.0239 (3)	
C6	0.15775 (8)	0.55115 (19)	0.26184 (9)	0.0220 (3)	
C7	0.13574 (8)	0.4223 (2)	0.19297 (9)	0.0254 (3)	
C8	0.08177 (8)	0.5102 (2)	0.11800 (9)	0.0264 (3)	
H8	0.0620	0.4171	0.0777	0.032*	
C9	0.01057 (9)	0.5906 (2)	0.13270 (9)	0.0285 (3)	
H9	−0.0244	0.6395	0.0820	0.034*	
C10	0.03877 (8)	0.7424 (2)	0.18927 (9)	0.0279 (3)	
H10A	0.0520	0.8416	0.1610	0.033*	
H10B	−0.0048	0.7799	0.2075	0.033*	
C11	0.20578 (9)	0.3494 (2)	0.17160 (9)	0.0275 (3)	
H11A	0.2421	0.2888	0.2176	0.033*	
H11B	0.1861	0.2628	0.1292	0.033*	
C12	0.25034 (9)	0.4894 (2)	0.14564 (9)	0.0281 (3)	
C13	0.20238 (9)	0.6325 (2)	0.09902 (9)	0.0266 (3)	
C14	0.12457 (9)	0.6423 (2)	0.08562 (9)	0.0259 (3)	
H14	0.0957	0.7353	0.0548	0.031*	
C15	0.08761 (10)	0.2737 (2)	0.21185 (10)	0.0316 (4)	
H15A	0.0736	0.1877	0.1688	0.038*	
H15B	0.1198	0.2137	0.2601	0.038*	
C16	0.01249 (10)	0.3444 (2)	0.22285 (10)	0.0330 (4)	
H16A	−0.0202	0.2457	0.2293	0.040*	
H16B	0.0267	0.4156	0.2710	0.040*	
C17	0.31222 (10)	0.6993 (2)	0.52708 (9)	0.0332 (4)	
H17A	0.3228	0.8142	0.5092	0.050*	
H17B	0.3590	0.6580	0.5686	0.050*	
H17C	0.2681	0.7079	0.5471	0.050*	
C18	0.20598 (10)	0.8932 (2)	0.03173 (10)	0.0355 (4)	
H18A	0.1646	0.8552	−0.0158	0.053*	
H18B	0.2431	0.9685	0.0177	0.053*	
H18C	0.1823	0.9579	0.0649	0.053*	
C19	−0.10438 (10)	0.5180 (3)	0.16953 (12)	0.0392 (4)	
H19A	−0.1362	0.4201	0.1767	0.059*	
H19B	−0.1352	0.5874	0.1245	0.059*	
H19C	−0.0890	0.5911	0.2164	0.059*	
C20	0.34415 (9)	0.3842 (2)	0.35220 (9)	0.0273 (3)	
H20A	0.3687	0.4621	0.3971	0.033*	
H20B	0.3512	0.4367	0.3051	0.033*	
C21	0.38241 (9)	0.2075 (2)	0.36728 (9)	0.0262 (3)	
C22	0.46433 (10)	0.1988 (2)	0.39458 (10)	0.0323 (4)	
H22	0.4942	0.3033	0.4049	0.039*	
C23	0.50269 (10)	0.0401 (3)	0.40683 (11)	0.0394 (4)	
H23	0.5585	0.0343	0.4250	0.047*	
C24	0.45800 (11)	−0.1091 (3)	0.39200 (12)	0.0398 (4)	
C25	0.37742 (11)	−0.1056 (2)	0.36530 (12)	0.0397 (4)	
H25	0.3480	−0.2107	0.3556	0.048*	
C26	0.33978 (10)	0.0545 (2)	0.35278 (11)	0.0335 (4)	
H26	0.2839	0.0590	0.3340	0.040*	
O2S	0.39668 (7)	0.7129 (2)	0.03873 (8)	0.0485 (4)	
H21S	0.4268	0.7898	0.0802	0.043 (6)*	
H22S	0.3668	0.6573	0.0628	0.131 (15)*	
O1S	0.5000	0.4796 (3)	0.0000	0.0471 (5)	
H11S	0.4712 (14)	0.555 (4)	0.0168 (15)	0.059 (7)*	

### Atomic displacement parameters (Å^2^)

**Table:**

	*U*^11^	*U*^22^	*U*^33^	*U*^12^	*U*^13^	*U*^23^
F1	0.0562 (6)	0.0372 (6)	0.0885 (9)	0.0217 (5)	0.0383 (6)	0.0200 (6)
N1	0.0288 (6)	0.0374 (8)	0.0294 (8)	−0.0071 (6)	0.0115 (5)	−0.0058 (6)
O1	0.0254 (5)	0.0206 (5)	0.0261 (6)	0.0017 (4)	0.0101 (4)	0.0017 (4)
O2	0.0308 (5)	0.0273 (5)	0.0195 (6)	0.0014 (4)	0.0045 (4)	−0.0017 (4)
O3	0.0355 (6)	0.0483 (8)	0.0397 (7)	0.0102 (5)	0.0205 (5)	0.0108 (6)
O4	0.0313 (5)	0.0316 (6)	0.0331 (6)	−0.0011 (5)	0.0158 (4)	0.0059 (5)
C1	0.0241 (6)	0.0198 (7)	0.0241 (8)	−0.0004 (5)	0.0117 (5)	0.0022 (6)
C2	0.0244 (7)	0.0244 (7)	0.0219 (8)	−0.0044 (6)	0.0088 (6)	0.0001 (6)
C3	0.0298 (7)	0.0260 (8)	0.0253 (8)	−0.0044 (6)	0.0113 (6)	−0.0051 (6)
C4	0.0258 (7)	0.0220 (7)	0.0299 (9)	0.0000 (6)	0.0117 (6)	−0.0004 (6)
C5	0.0223 (6)	0.0252 (8)	0.0251 (8)	−0.0033 (6)	0.0091 (6)	0.0035 (6)
C6	0.0248 (7)	0.0221 (7)	0.0215 (8)	−0.0046 (6)	0.0110 (6)	0.0005 (6)
C7	0.0310 (7)	0.0261 (8)	0.0194 (8)	−0.0040 (6)	0.0086 (6)	−0.0019 (6)
C8	0.0290 (7)	0.0287 (8)	0.0208 (8)	−0.0043 (6)	0.0072 (6)	−0.0022 (6)
C9	0.0263 (7)	0.0342 (9)	0.0230 (9)	−0.0044 (7)	0.0056 (6)	−0.0004 (7)
C10	0.0259 (7)	0.0294 (8)	0.0271 (9)	0.0014 (6)	0.0070 (6)	0.0018 (6)
C11	0.0398 (8)	0.0236 (8)	0.0207 (8)	0.0012 (6)	0.0120 (6)	−0.0038 (6)
C12	0.0346 (8)	0.0314 (9)	0.0214 (8)	0.0022 (7)	0.0138 (6)	−0.0038 (6)
C13	0.0330 (7)	0.0301 (8)	0.0188 (8)	−0.0025 (6)	0.0113 (6)	−0.0019 (6)
C14	0.0311 (7)	0.0286 (8)	0.0165 (8)	−0.0042 (6)	0.0060 (6)	−0.0013 (6)
C15	0.0391 (8)	0.0270 (8)	0.0289 (9)	−0.0083 (7)	0.0115 (7)	−0.0035 (7)
C16	0.0371 (8)	0.0350 (9)	0.0296 (9)	−0.0130 (7)	0.0145 (6)	−0.0028 (7)
C17	0.0426 (9)	0.0300 (8)	0.0216 (9)	−0.0040 (7)	0.0034 (6)	−0.0033 (6)
C18	0.0374 (8)	0.0350 (9)	0.0336 (10)	−0.0049 (8)	0.0109 (7)	0.0074 (7)
C19	0.0319 (8)	0.0491 (11)	0.0393 (10)	−0.0082 (8)	0.0154 (7)	−0.0081 (8)
C20	0.0284 (7)	0.0261 (8)	0.0310 (9)	−0.0007 (6)	0.0146 (6)	0.0020 (7)
C21	0.0332 (7)	0.0289 (8)	0.0215 (8)	0.0050 (6)	0.0156 (6)	0.0034 (6)
C22	0.0334 (8)	0.0355 (9)	0.0283 (9)	−0.0011 (7)	0.0104 (6)	−0.0009 (7)
C23	0.0343 (9)	0.0478 (11)	0.0369 (10)	0.0089 (8)	0.0128 (7)	0.0037 (8)
C24	0.0459 (9)	0.0355 (9)	0.0466 (11)	0.0143 (8)	0.0267 (8)	0.0149 (8)
C25	0.0448 (9)	0.0258 (8)	0.0596 (12)	0.0024 (8)	0.0322 (9)	0.0078 (8)
C26	0.0318 (8)	0.0301 (9)	0.0446 (10)	0.0029 (7)	0.0208 (7)	0.0054 (7)
O2S	0.0406 (6)	0.0605 (9)	0.0520 (8)	−0.0194 (7)	0.0256 (6)	−0.0300 (7)
O1S	0.0402 (10)	0.0401 (11)	0.0665 (14)	0.000	0.0251 (10)	0.000

### Geometric parameters (Å, °)

**Table:**

F1—C24	1.360 (2)	C12—C13	1.479 (2)
N1—C19	1.469 (2)	C13—C14	1.343 (2)
N1—C16	1.471 (2)	C14—H14	0.9500
N1—C9	1.476 (2)	C15—C16	1.531 (2)
O1—C1	1.3815 (18)	C15—H15A	0.9900
O1—C20	1.4438 (17)	C15—H15B	0.9900
O2—C2	1.3725 (18)	C16—H16A	0.9900
O2—C17	1.430 (2)	C16—H16B	0.9900
O3—C12	1.2233 (19)	C17—H17A	0.9800
O4—C13	1.3585 (19)	C17—H17B	0.9800
O4—C18	1.425 (2)	C17—H17C	0.9800
C1—C6	1.406 (2)	C18—H18A	0.9800
C1—C2	1.411 (2)	C18—H18B	0.9800
C2—C3	1.383 (2)	C18—H18C	0.9800
C3—C4	1.380 (2)	C19—H19A	0.9800
C3—H3	0.9500	C19—H19B	0.9800
C4—C5	1.379 (2)	C19—H19C	0.9800
C4—H4	0.9500	C20—C21	1.505 (2)
C5—C6	1.420 (2)	C20—H20A	0.9900
C5—C10	1.517 (2)	C20—H20B	0.9900
C6—C7	1.540 (2)	C21—C26	1.381 (2)
C7—C15	1.539 (2)	C21—C22	1.394 (2)
C7—C8	1.542 (2)	C22—C23	1.381 (3)
C7—C11	1.543 (2)	C22—H22	0.9500
C8—C14	1.506 (2)	C23—C24	1.375 (3)
C8—C9	1.525 (2)	C23—H23	0.9500
C8—H8	1.0000	C24—C25	1.370 (3)
C9—C10	1.525 (2)	C25—C26	1.386 (3)
C9—H9	1.0000	C25—H25	0.9500
C10—H10A	0.9900	C26—H26	0.9500
C10—H10B	0.9900	O2S—H21S	0.9694
C11—C12	1.507 (2)	O2S—H22S	0.9051
C11—H11A	0.9900	O1S—H11S	0.90 (3)
C11—H11B	0.9900		
			
C19—N1—C16	109.97 (14)	O4—C13—C12	111.83 (13)
C19—N1—C9	112.02 (14)	C13—C14—C8	122.02 (15)
C16—N1—C9	115.49 (12)	C13—C14—H14	119.0
C1—O1—C20	115.64 (11)	C8—C14—H14	119.0
C2—O2—C17	116.41 (12)	C16—C15—C7	110.70 (13)
C13—O4—C18	115.67 (12)	C16—C15—H15A	109.5
O1—C1—C6	120.15 (13)	C7—C15—H15A	109.5
O1—C1—C2	118.52 (12)	C16—C15—H15B	109.5
C6—C1—C2	121.03 (14)	C7—C15—H15B	109.5
O2—C2—C3	123.80 (14)	H15A—C15—H15B	108.1
O2—C2—C1	116.07 (13)	N1—C16—C15	111.84 (13)
C3—C2—C1	120.13 (13)	N1—C16—H16A	109.2
C4—C3—C2	118.93 (14)	C15—C16—H16A	109.2
C4—C3—H3	120.5	N1—C16—H16B	109.2
C2—C3—H3	120.5	C15—C16—H16B	109.2
C5—C4—C3	122.39 (14)	H16A—C16—H16B	107.9
C5—C4—H4	118.8	O2—C17—H17A	109.5
C3—C4—H4	118.8	O2—C17—H17B	109.5
C4—C5—C6	120.01 (13)	H17A—C17—H17B	109.5
C4—C5—C10	117.71 (14)	O2—C17—H17C	109.5
C6—C5—C10	122.25 (14)	H17A—C17—H17C	109.5
C1—C6—C5	117.47 (13)	H17B—C17—H17C	109.5
C1—C6—C7	121.96 (13)	O4—C18—H18A	109.5
C5—C6—C7	120.11 (13)	O4—C18—H18B	109.5
C15—C7—C6	107.95 (12)	H18A—C18—H18B	109.5
C15—C7—C8	106.49 (12)	O4—C18—H18C	109.5
C6—C7—C8	110.68 (13)	H18A—C18—H18C	109.5
C15—C7—C11	110.81 (13)	H18B—C18—H18C	109.5
C6—C7—C11	115.14 (12)	N1—C19—H19A	109.5
C8—C7—C11	105.44 (12)	N1—C19—H19B	109.5
C14—C8—C9	111.39 (13)	H19A—C19—H19B	109.5
C14—C8—C7	112.60 (12)	N1—C19—H19C	109.5
C9—C8—C7	110.14 (12)	H19A—C19—H19C	109.5
C14—C8—H8	107.5	H19B—C19—H19C	109.5
C9—C8—H8	107.5	O1—C20—C21	108.57 (12)
C7—C8—H8	107.5	O1—C20—H20A	110.0
N1—C9—C8	108.72 (13)	C21—C20—H20A	110.0
N1—C9—C10	116.85 (14)	O1—C20—H20B	110.0
C8—C9—C10	108.37 (12)	C21—C20—H20B	110.0
N1—C9—H9	107.5	H20A—C20—H20B	108.4
C8—C9—H9	107.5	C26—C21—C22	118.90 (15)
C10—C9—H9	107.5	C26—C21—C20	122.68 (13)
C5—C10—C9	113.63 (14)	C22—C21—C20	118.39 (15)
C5—C10—H10A	108.8	C23—C22—C21	120.94 (16)
C9—C10—H10A	108.8	C23—C22—H22	119.5
C5—C10—H10B	108.8	C21—C22—H22	119.5
C9—C10—H10B	108.8	C24—C23—C22	118.28 (15)
H10A—C10—H10B	107.7	C24—C23—H23	120.9
C12—C11—C7	112.63 (13)	C22—C23—H23	120.9
C12—C11—H11A	109.1	F1—C24—C25	118.55 (17)
C7—C11—H11A	109.1	F1—C24—C23	119.01 (15)
C12—C11—H11B	109.1	C25—C24—C23	122.44 (17)
C7—C11—H11B	109.1	C24—C25—C26	118.62 (17)
H11A—C11—H11B	107.8	C24—C25—H25	120.7
O3—C12—C13	121.36 (15)	C26—C25—H25	120.7
O3—C12—C11	122.58 (15)	C21—C26—C25	120.83 (15)
C13—C12—C11	115.97 (13)	C21—C26—H26	119.6
C14—C13—O4	126.68 (15)	C25—C26—H26	119.6
C14—C13—C12	121.49 (14)	H21S—O2S—H22S	100.4
			
C20—O1—C1—C6	117.00 (14)	C7—C8—C9—C10	67.54 (17)
C20—O1—C1—C2	−69.19 (16)	C4—C5—C10—C9	−161.88 (13)
C17—O2—C2—C3	−6.5 (2)	C6—C5—C10—C9	16.3 (2)
C17—O2—C2—C1	174.39 (13)	N1—C9—C10—C5	76.09 (17)
O1—C1—C2—O2	4.70 (18)	C8—C9—C10—C5	−47.08 (17)
C6—C1—C2—O2	178.46 (12)	C15—C7—C11—C12	174.84 (13)
O1—C1—C2—C3	−174.47 (12)	C6—C7—C11—C12	−62.30 (18)
C6—C1—C2—C3	−0.7 (2)	C8—C7—C11—C12	59.99 (16)
O2—C2—C3—C4	−177.53 (14)	C7—C11—C12—O3	146.72 (15)
C1—C2—C3—C4	1.6 (2)	C7—C11—C12—C13	−36.64 (19)
C2—C3—C4—C5	−0.4 (2)	C18—O4—C13—C14	4.3 (2)
C3—C4—C5—C6	−1.7 (2)	C18—O4—C13—C12	−176.15 (14)
C3—C4—C5—C10	176.50 (14)	O3—C12—C13—C14	−178.85 (14)
O1—C1—C6—C5	172.34 (12)	C11—C12—C13—C14	4.5 (2)
C2—C1—C6—C5	−1.31 (19)	O3—C12—C13—O4	1.6 (2)
O1—C1—C6—C7	0.16 (19)	C11—C12—C13—O4	−175.12 (12)
C2—C1—C6—C7	−173.49 (13)	O4—C13—C14—C8	−179.60 (15)
C4—C5—C6—C1	2.50 (19)	C12—C13—C14—C8	0.9 (2)
C10—C5—C6—C1	−175.63 (13)	C9—C8—C14—C13	150.28 (14)
C4—C5—C6—C7	174.83 (13)	C7—C8—C14—C13	26.0 (2)
C10—C5—C6—C7	−3.3 (2)	C6—C7—C15—C16	60.26 (16)
C1—C6—C7—C15	77.55 (16)	C8—C7—C15—C16	−58.62 (17)
C5—C6—C7—C15	−94.43 (16)	C11—C7—C15—C16	−172.80 (13)
C1—C6—C7—C8	−166.27 (12)	C19—N1—C16—C15	−179.28 (13)
C5—C6—C7—C8	21.76 (18)	C9—N1—C16—C15	−51.32 (19)
C1—C6—C7—C11	−46.83 (19)	C7—C15—C16—N1	52.98 (18)
C5—C6—C7—C11	141.20 (14)	C1—O1—C20—C21	175.38 (12)
C15—C7—C8—C14	−171.75 (13)	O1—C20—C21—C26	14.3 (2)
C6—C7—C8—C14	71.16 (16)	O1—C20—C21—C22	−167.91 (13)
C11—C7—C8—C14	−53.97 (17)	C26—C21—C22—C23	0.2 (2)
C15—C7—C8—C9	63.25 (16)	C20—C21—C22—C23	−177.70 (16)
C6—C7—C8—C9	−53.84 (16)	C21—C22—C23—C24	−0.5 (3)
C11—C7—C8—C9	−178.96 (12)	C22—C23—C24—F1	179.61 (18)
C19—N1—C9—C8	−178.64 (13)	C22—C23—C24—C25	0.4 (3)
C16—N1—C9—C8	54.42 (18)	F1—C24—C25—C26	−179.18 (17)
C19—N1—C9—C10	58.37 (18)	C23—C24—C25—C26	0.0 (3)
C16—N1—C9—C10	−68.57 (18)	C22—C21—C26—C25	0.2 (3)
C14—C8—C9—N1	173.90 (13)	C20—C21—C26—C25	178.05 (17)
C7—C8—C9—N1	−60.41 (17)	C24—C25—C26—C21	−0.4 (3)
C14—C8—C9—C10	−58.15 (17)		

### Hydrogen-bond geometry (Å, °)

**Table:**

*D*—H···*A*	*D*—H	H···*A*	*D*···*A*	*D*—H···*A*
O2S—H22S···O4	0.91	2.33	2.9805 (16)	128
O2S—H22S···O3	0.91	2.54	3.417 (2)	164
O1S—H11S···O2S	0.90 (3)	1.94 (3)	2.8342 (19)	172 (2)
O2S—H21S···N1^i^	0.97	1.81	2.7736 (19)	170

Symmetry codes: (i) *x*+1/2, *y*+1/2, *z*.
